# Diagnostic value of non-contrast brain computed tomography in the evaluation of acute cerebral venous thrombosis

**DOI:** 10.1038/s41598-020-57867-1

**Published:** 2020-01-21

**Authors:** Shamim Tayyebi, Reza Akhavan, Majid Shams, Maryam Salehi, Donya Farrokh, Farhad Yousefi, Bita Abbasi

**Affiliations:** 1 Department of Radiology, Faculty of Medicine, Mashhad University of Medical Sciences, Mashhad, Iran; 2 Department of Emergency Medicine, Faculty of Medicine, Mashhad University of Medical Sciences, Mashhad, Iran; 3 Department of Community Medicine, Faculty of Medicine, Mashhad University of Medical Sciences, Mashhad, Iran

**Keywords:** Stroke, Cerebrovascular disorders

## Abstract

Acute cerebral vein thrombosis is usually seen as increased attenuation in brain non-contrast computed tomography. It is so helpful to define measurable parameters for subjective evaluation of sinus thrombosis in non-enhanced brain computed tomography, especially where advanced neuroimaging techniques are not available. The purpose of this study is to evaluate the diagnostic value of venobasilar attenuation ratio and venobasilar attenuation difference in the evaluation of acute cerebral venous sinuous thrombosis in non-enhanced brain CT scan. Fifty confirmed cases of acute cerebral vein thrombosis were sex- and age-matched with 73 subjects who did not have the condition. Average venous sinus attenuation, Hounsfield to hematocrit ratio, basilar artery density, venobasilar attenuation ratio and venobasilar attenuation difference were measured. Mean attenuation was 65.8 in thrombosed and 44.9 in non-thrombosed sinuses (P < 0.0001). A cutoff absolute sinus attentuaion of 61 HU led to a sensitivity of 82%, specificity of 100% and accuracy of 92%. A cutoff ratio of 1.4 for venobasilar ratio led to a sensitivity of 100%, specificity of 78% and accuracy of 87%. A cut-off value of 24 for venobasilar difference resulted in the sensitivity of 80%, specificity of 100% and accuracy of 92%. The additional measurement of venous sinus and basilar artery attenuations and calculation of venobasilar ratio and difference can increase the sensitivity and specificity of NCCT in the diagnosis of acute CVST.

## Introduction

Cerebral venous sinus thrombosis (CVST) is responsible for about 0.5–1% of all strokes and occurs in about 2–5 patients per million every year^1,2^. Patients exhibit a wide range of non-specific signs and symptoms, including -but not limited to - headache (the most common symptom), focal neurologic deficits, seizure and impaired level of consciousness. So, the diagnosis is usually not suspected at the initial presentation^3,4^. NCCT may be the only modality available in low resource settings^5^. The elevated density of the venous sinuses (dense sinus sign or cord sign) is the only direct sign of acute CVT in brain NCCT^6^. It has been shown in several studies that blood density within dural veins correlates with hematocrit level^7–10^, and polycythemia is considered as a mimicker of CVST^11,12^. It seems rational to define quantitative criteria for more accurate diagnosis and reduce the false positive results. The aim of this study is to evaluate NCCT in patients with and without acute CVST and determine the diagnostic value of venous sinus density, Hounsfield-hematocrit (H:H) ratio, veno-basilar (VB) ratio and VB difference in the evaluation of acute CVST.

## Materials and Methods

### Patient selection

The ethics committee of Mashhad university of medical sciences approved the study (approval ID: IR.MUMS.MEDICAL.REC.1398.233) and waived the need for informed consent.

In this retrospective observational study, we sought the archive of our academic hospital which is a first-level and referral neurology center, to compile the database of patients with and without acute CVST. The initial brain NCCT and MRV examinations of 500 patients with acute neurologic presentations (presenting from April 2010 to December 2017) were evaluated and 50 patients with confirmed CVST were sex- and age-matched with 73 patients without this condition. These studies were loaded in a diagnostic station in alphabetical order, anonymized and read by an experienced neuroradiologist who was blind to the patients’ clinical status.

Inclusion criteria included:

1. Adult patients ≥18-year-old with no prior history of cerebral venous thrombosis admitted for acute neurologic symptoms (less than 7 days).
2. NCCT at the admission and brain contrast-enhanced MRV performed within 72 hours of admission.
3. Confirmation of cerebral venous thrombosis with contrast enhanced MRV as the reference standard performed within 10 days of the NCCT.

The exclusion criteria were intracranial hemorrhage, skull fracture in the vicinity of venous sinuses, signs of increased intracranial pressure such as hydrocephalus, cerebral edema (as defined by sulcal effacement or decreased supratentorial attenuation) or intra-and extra-axial mass, recent cerebral surgery and history of any suspicious clinical symptoms of venous thrombosis lasting more than 7 days before the NCCT. Patients who had received blood transfusion in the time period between hematocrit level assessment and NCCT, were also excluded.

### Imaging review

MRV examinations were reviewed by two expert neuroradiologists (that were blind to the results of CT densitometry in venous sinuses), and the present or absent of CVST was defined as the consensus of them. In cases of confirmed CVST, location of the thrombus was recorded.

The anonymized NCCT of patients and controls were randomly evaluated using Osirix-MD™ (version 10.0.1) software on medical monitors by a neuroradiology staff. The reader was allowed to routine adjustment of image contrast and magnification and was asked to analyze CT slices with the least beam hardening artifact.

**Figure 1:**
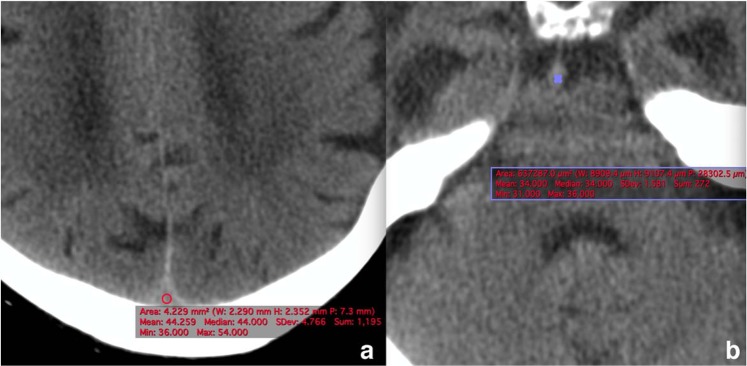
Sinus attenuation measurement at the level of superior sagittal sinus (**a**) and basilar artery (**b**). The ROI was placed at the center of the vascular structure, and was not in contact with its wall.

Venous sinuous structures were categorized into following segments: superior sagittal, torcula herophili, left sigmoid sinus and right sigmoid sinuses. The reader was asked to measure the CT attenuation of each venous segment, using an oval region of interest (ROI). The ROI was placed at the center of the venous sinus, and was not in contact with the sinus wall (Fig. 1a). The measurement was performed two times for each segment, and the mean attenuation value was documented in Hounsfield Unit (HU). Average HU was defined as the mean of four sinus segments in the control group, and the mean density of involved sinus segments in the case group. The H: H ratio was calculated by dividing average HU by the HCT level. An ROI was also placed within a non-calcified portion of basilar artery in a slice that it was seen end-on as a round structure anterior to the pons and blood attenuation within this arterial structure was documented as basilar artery density (Fig. 1b). VB ratio was calculated by dividing average HU by basilar artery density. VB difference was calculated by subtracting the basilar artery attenuation from the mean sinus density.

### Statistical analysis

Statistical analyses were performed using SPSS v.21 for Windows (SPSS, Chicago, Illinois). Continuous variables are presented as mean ± SD, unless specified otherwise. Absolute attenuation and ratios of thrombosed and non-thrombosed venous sinuses were compared via Mann Whitney test. For average HU, H:H ratio VB ratio and VB difference, receiver operating characteristic (ROC) curves were used to assess their values in identifying sinus thrombosis and find optimal cutoff values, for which sensitivity, specificity, and accuracy were reported. A *P* value of <0.05 is considered to indicate a statistically significant result.

### Ethical approval

The Ethics Committee of Mashhad university of medical sciences approved the study and waived the need for informed consent as part of the study approval.

### Informed consent

Informed consent was waived by the Ethics Committee of Mashhad University of Medical Sciences as part of the study approval.

## Results

### Descriptive statistics

There were 50 patients in the case (and 73 patients in the control group. There were no statistically significant differences in the sex (*P* = 0.52, Chi-square test) and mean age (*P* = 0.76) of the two groups. The Kappa agreement between the two observers was 0.93.

HCT levels ranged from 26.5% to 58.9% (mean, 39.8%) in the patient, and 15.0% to 61.2% (mean, 37.4%) in the control group, and showed no significant difference between the two groups (*P* = 0.09). BUN to creatinine ratio was calculated and used as a criterion for dehydration^13^. There was no statistically significant difference in the BUN/Cr of the two groups (*P* = 0.55).

### Density measurement parameters and optimal cutoffs

**Figure 2:**
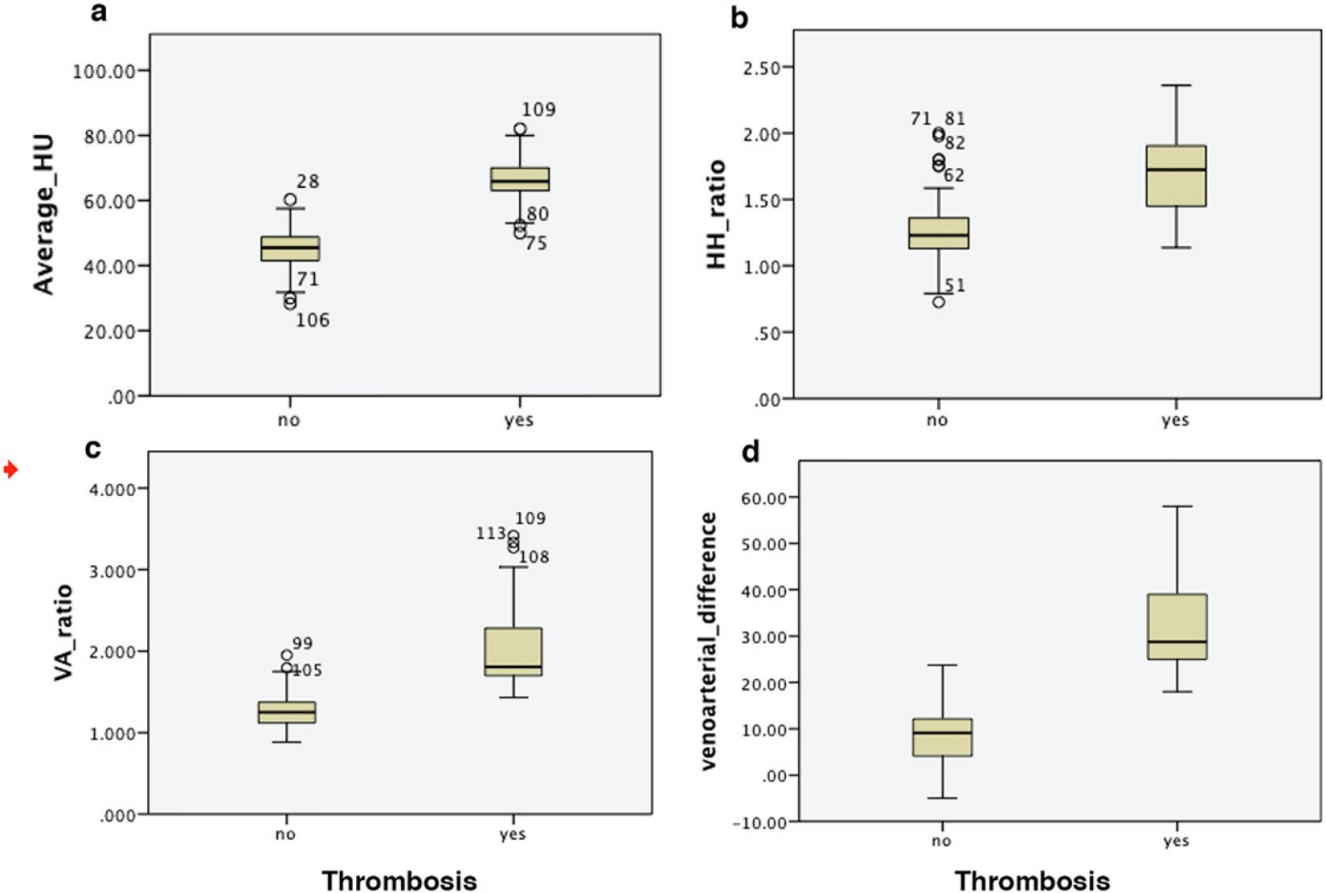
Mean average HU was significantly different in patients with acute CVST (65.84 ± 6.44 HU) compared with control participants (52.8 ± 6.7 HU) (**a**). The mean H:H ratio showed values of 1.33 ± 0.12 in subjects without CVST, and 1.69 ± 0.32 in those with CVST (P < 0.0001) (**b**). Mean VA ratio showed values of 1.33 ± 0.12 in subjects without CVST, and 2.07 ± 0.55 in patients with CVST (P < 0.0001) (**c**) Mean VA difference was in 8.9 (±6.5) subjects without CVST and 32.26 (±9.32) in patients with CVST (P < 0.0001) (**d**).

**Figure 3:**
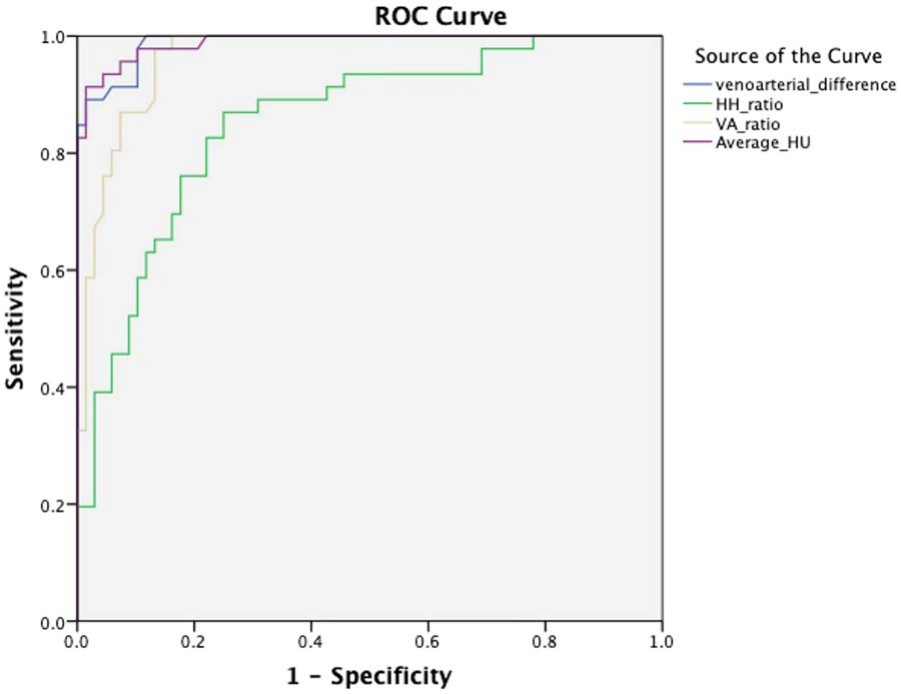
ROC curves comparing the difference between average HU, H:H ratio, VA ratio and VA difference measurement in evaluating acute CVST.

The mean attenuation of sinuses with thrombosis was higher than the patent sinuses: 66 HU (95% CI 59.6–72.4) for thrombosed sinuses vs. 45 (95% CI 38.8–51.2) for patent sinuses (*P* < 0.0001) (Fig. 2). The receiver operating characteristic (ROC) analysis of average HU showed an area under the curve (AUC) of 0.990 and an optimal cutoff value of 61 HU, leading to a sensitivity of 82%, specificity of 100% and accuracy of 92% (Fig. 3). Results of attenuation calculation in vascular structures are summarized in Table 1.

**Table 1:** HU values for arterial and venous structures in both patient and control groups.

Mean values	Patient group	Control group	P value
Basilar artery density	33.59	35.04	0.051
Average venous HU	65.8	44.9	0.000
VA ratio	2.06	1.3	0.000
VA difference	32.26	8.9	0.000

HU: Hounsfield Unit, VA: venoarterial.

H: H ratio in the thrombosed sinuses was significantly higher than the non-thrombosed sinuses (1.7 ± 0.3 and 1.2 ± 0.2 respectively) (*P* < *0.0001*). The ROC analysis of the H: H ratio showed an AUC of 0.87 and an optimal cutoff value of 1.5, leading to a sensitivity of 66%, specificity of 85% and accuracy of 72% (Fig. 3).

The mean VB ratio ranged from 1.43 to 3.4 (mean, 2.1 ± 0.5) in the thromboses sinuses and 0.9 to 1.9 (mean, 1.3 ± 0.2) in the patent sinuses (*P* < 0.0001). In the thrombosed sinuses, the VB difference reached a mean value of 32.3 (±9.3 SD) HU that was significantly higher than the non-thromboses venous sinuses (mean 8.9 ± 6.5 HU) (*P* < 0.0001) (Fig. 2). The receiver operating characteristic (ROC) analysis of VB ratio showed an area under the curve of 0.965 and an optimal cutoff ratio of 1.43, leading to a sensitivity of 100%, specificity of 78% and accuracy of 87%. The ROC analysis of VB difference showed an AUC of 0.989, and a cut-off value of 24 resulted in the sensitivity of 80%, specificity of 100% and accuracy of 92% (Fig. 3) (Table 2).

**Table 2:** Summary of sensitivity and specificity data for quantitative parameters in the evaluation of acute cerebral venous thrombosis.

Criterion	Sensitivity	Specificity	Accuracy
Average HU > 61	82%	100%	92%
H:H ratio > 1.5	66%	88%	72%
VA ratio > 1.43	100%	78%	87%
VA difference > 24	80%	100%	92%

HU: Hounsfield Unit, H:H ratio: Hounsfield Hematocrit ratio, VA: venoarterial.

## Discussion

Acute thrombosis within the vessel lumen is usually seen as hyperattenuating material in NCCT performed in the acute phase of the disease. The increased attenuation, usually attributed to the decreased amount of water in the retracted clot and increased concentration of red blood cells and hemoglobin^4,14^, is known as cord sign or dense triangle sign. The sensitivity of this finding is reported as 63–73% in different studies^4,15,16^. Quantitative measurement of attenuation is believed to be more accurate than subjective evaluation, as it can appreciate moderate increases in mean sinus density^15^. Our findings suggest that a cutoff value of 61 can discriminate between venous structures with and without acute CVST with a sensitivity of 82% and specificity of 100%. Buyck *et al*. found the similar cutoff value of 61 in their study on 20 patients with acute CVST^7^.

Elevated serum hematocrit can result in increased sinus attenuation and false positive readings^11,12^. Black *et al*. were the first who proposed H:H ratio as a means for normalizing sinus density according to the patient’s hematocrit level. They found a mean H:H value of 2.2 in 8 patients with CVST and 1.4 in subjects without CVST and suggested 1.8 as the threshold for CVST^8^. Buyck *et al*. suggested a cutoff value of 1.5 for H:H to diagnose acute CVST. In the current study, we suggest the cutoff value of 1.5 as a threshold to suspect the presence of thrombosis, but ROC curve analysis returned a better diagnostic performance for absolute sinus attenuation than H:H ratio.

Our study proposed using VB ratio and VB difference as more accurate normalized attenuation parameters. In contrast to H:H ratio, these factors do not require the acquisition of blood work and can be readily calculated directly from the NCCT. Both parameters showed significant differences between thrombosed and patent sinuses. This suggests that there might be a hidden quantitative criterion for diagnosing acute CVST in the NCCT that is not apparent on visual inspection. On the ROC curve analysis VB ratio had a sensitivity of 100% and VB difference had a specificity of 100%, making them complementary parameters in diagnosing acute CVST using just one additional measurement. In terms of specificity, average sinus attenuation also returned a specificity of 100% on ROC curve analysis, suggesting a better performance for absolute attenuation measurement than H:H ratio calculation. This may be justified by taking into account that H:H ratio considers just one inherent factor that effects blood attenuation in the NCCT (i.e. hemoatocrite level), but VB difference and VB ratio normalize the sinous venous attenuation using another blood containing structure (i.e. basilar artery). This makes all the known and unknown factors that are responsible for blood attenuation be part of the game. However, our study is performed retrospectively and needs to be validated in a prospective study.

There are some limitations in our study that need to be mentioned. Dural sinuses are located adjacent to the skull, and the partial volume effects could have caused false-positive hyperattenuation in the venous sinuses. Although all reader were blind to the clinical data, the presence of indirect signs of sinus thrombosis in the NCCT could have resulted in unwanted selection bias.

## Conclusions

NCCT is globally the first requested modality in the setting of acute neurologic symptoms. We found that quantitative measurement of venous sinus and basilar artery attenuations and calculation of VB ratio and VB difference improved the diagnostic performance of NCCT in diagnosing acute CVST over visual inspection. VB ratio > 1.4 is the optimal cutoff for sensitivity, and VB difference > 24 or absolute sinus attenuation > 61 are optimal cutoffs for specificity. Validation of these cutoffs needs prospective cohort studies.

## Data Availability

The SPSS file is available on personal demand.
